# A tetra­silver(I)ditungsten(VI) cluster with sulfide and bis­(diphenyl­phosphino)methane ligands

**DOI:** 10.1107/S1600536810034197

**Published:** 2010-09-04

**Authors:** Rong Wang, Li-Li Song, Ke-Yi Hu, Qiong-Hua Jin, Cun-Lin Zhang

**Affiliations:** a Department of Chemistry, Capital Normal University, Beijing 100048, People’s Republic of China; b Beijing Key Laboratory for Terahertz Spectroscopy and Imaging, Key Laboratory of Terahertz Optoelectronics, Ministry of Education, Capital Normal University, Beijing 100048, People’s Republic of China

## Abstract

The asymmetric unit of the title complex, [Ag_4_W_2_S_8_(C_25_H_22_P_2_)_3_]·2C_3_H_7_NO, tris­[μ_2_-bis­(diphenyl­phosphino)meth­ane]-3:6κ^2^ *P*:*P*′;4:5κ^2^ *P*:*P*′;5:6κ^2^ *P*:*P*′-μ_5_-sulfido-2:3:4:5:6κ^5^ *S*-μ_3_-sulfido-1:3:4κ^3^ *S*-tetra-μ_2_-sulfido-1:3κ^2^ *S*;1:4κ^2^ *S*;2:5κ^2^ *S*;2:6κ^2^ *S*-disulfido-1κ*S*,2κ*S*-tetra­silver(I)ditungsten(VI) *N*,*N*-dimethyl­formamide disolvate, contains two [WS_4_]^2−^ anions, four silver cations, three bidentate–bridging bis­(diphenyl­phosphino)methane (dppm) ligands and two *N*,*N*-dimethyl­formamide (DMF) solvent mol­ecules. The coordination geometry of each Ag atom is distorted tetra­hedral. Two Ag ions are coordinated by μ_2_-S and μ_5_-S atoms, and by two P atoms from two dppm ligands, while the other two Ag atoms are coordinated by μ_2_-S, μ_3_-S and μ_5_-S atoms, and by one P atom from a dppm ligand.

## Related literature

For related structures, see: Yu *et al.* (2001 ▶). For general background to Mo(W)—Cu(Ag)—S clusters derived from tetra­thio­tungstate and tetra­thio­molybdate [*M*S_4_]^2−^(*M* = Mo, W) synthons, see: George *et al.*(2000 ▶, 2003 ▶); Hong *et al.* (1997 ▶); Lang *et al.* (2006 ▶); Niu *et al.* (2005 ▶); Ren *et al.* (2006 ▶); Shi *et al.* (1995 ▶); Yu *et al.* (2001 ▶); Zhang *et al.* (2000 ▶, 2004 ▶).
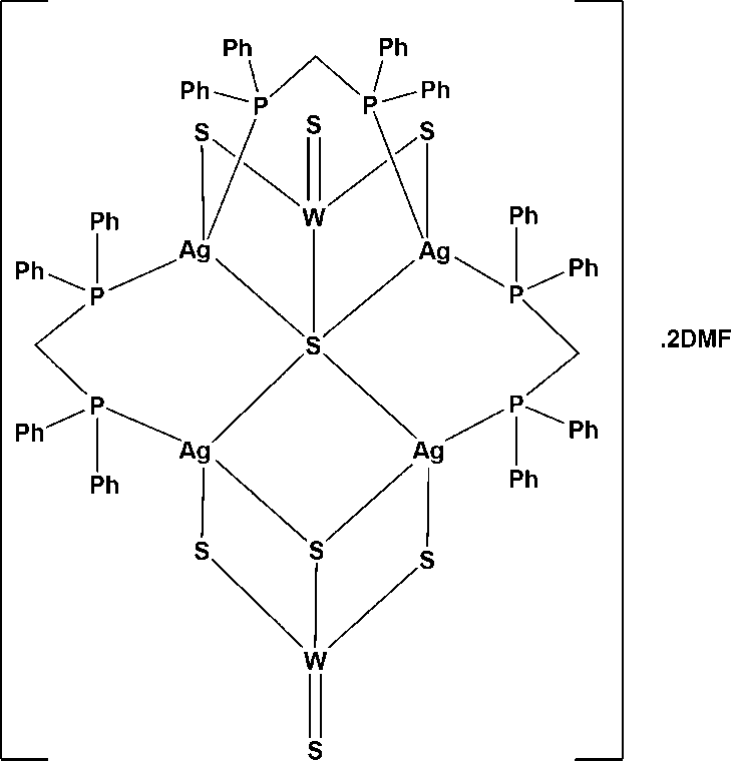


## Experimental

### 

#### Crystal data

[Ag_4_W_2_S_8_(C_25_H_22_P_2_)_3_]·2C_3_H_7_NO
*M* *_r_* = 2354.95
Monoclinic, 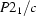

*a* = 22.931 (2) Å
*b* = 14.0395 (12) Å
*c* = 27.855 (3) Å
β = 107.224 (1)°
*V* = 8565.6 (14) Å^3^
*Z* = 4
Mo *K*α radiation
μ = 3.93 mm^−1^
*T* = 93 K
0.47 × 0.43 × 0.43 mm

#### Data collection

Rigaku R-AXIS RAPID diffractometer
Absorption correction: multi-scan (*ABSCOR*; Higashi, 1995 ▶) *T*_min_ = 0.262, *T*_max_ = 0.281
69256 measured reflections
19520 independent reflections
18777 reflections with *I* > 2σ(*I*)
*R*_int_ = 0.045

#### Refinement

*R*[*F* ^2^ > 2σ(*F* ^2^)] = 0.039
*wR*(*F* ^2^) = 0.089
*S* = 1.14
19520 reflections
950 parameters
H-atom parameters constrained
Δρ_max_ = 1.20 e Å^−3^
Δρ_min_ = −1.36 e Å^−3^

### 

Data collection: *RAPID-AUTO* (Rigaku, 2004 ▶); cell refinement: *RAPID-AUTO*; data reduction: *RAPID-AUTO*; program(s) used to solve structure: *SHELXS97* (Sheldrick, 2008 ▶); program(s) used to refine structure: *SHELXL97* (Sheldrick, 2008 ▶); molecular graphics: *ORTEPIII* (Burnett & Johnson, 1996 ▶) and *ORTEP-3 for Windows* (Farrugia, 1997 ▶); software used to prepare material for publication: *SHELXL97*.

## Supplementary Material

Crystal structure: contains datablocks global, I. DOI: 10.1107/S1600536810034197/dn2590sup1.cif

Structure factors: contains datablocks I. DOI: 10.1107/S1600536810034197/dn2590Isup2.hkl

Additional supplementary materials:  crystallographic information; 3D view; checkCIF report

## Figures and Tables

**Fig. 1.**
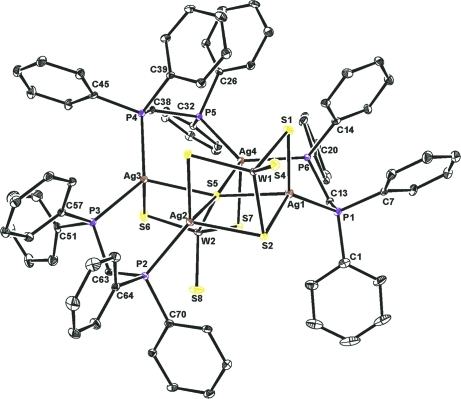
Molecular view of compound I with the atom labeling scheme. Ellipsoids are drawn at the 30% probability level. H atoms have been omitted for clarity.

**Fig. 2.**
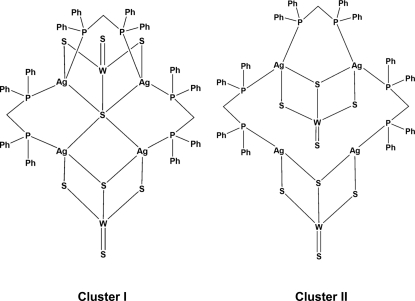
Comparison of the two isomers [(WS4)2Ag4(dppm)3] I and II.

## supplementary crystallographic information

### Comment

In the recent years, Mo(W)—Cu(Ag)—S clusters derived from the well known synthons tetrathiotungstate and tetrathiomolybdate([MS_4_]^2-^(*M*=Mo, W)) have been extensively investigated due to their rich structural chemistry (Zhang *et al.*, 2000; Zhang *et al.*, 2004; Lang *et al.*, 2006; Ren *et al.*, 2006) and potential applications in biological systems (George *et al.*, 2000; George *et al.* 2003), and optical materials (Shi *et al.*, 1995; Hong *et al.*, 1997; Niu *et al.*, 2005). Herein a new complex [(WS_4_)_2_Ag_4_(dppm)_3_].2DMF (1) [dppm=bis(diphenylphosphino)methane,DMF=*N*,*N*-dimethylformamide] is reported.

The asymmetric unit contains two [WS_4_]^2-^ anions, four silver cations, three bridging dppm ligands and two DMF solvent molecules(Fig. 1). One [WS_4_]^2-^ anion bridges two Ag atoms whereas the other [WS_4_]^2-^ anion bridges four Ag atoms. In both anions three sulfur atoms are coordinating to silver atoms whereas the W=S unit remaining intact. There are three kinds of bridging S atom: µ_2_-S (atoms S1, S3, S6 and S7, each is bonded to one W and one Ag atom), µ_3_-S (S2, bonded to W1, Ag1 and Ag2) and µ_5_-S (S5, bonded to W2 and four Ag atoms).

In complex 1, Ag1 and Ag2 are coordinated by one P atom from one dppm ligand, one µ_2_-S atom, one µ_3_-S atom and one µ_5_-S, while Ag3 and Ag4 are coordinated by two P atoms from two dppm ligands, one µ_2_-S atom and one µ_5_-S atom. It is noticeable that the coordination mode of µ_5_-S bonding to mixed metal ions is unique.

In two [WS_4_]^2-^ units, the average length of W-µ_2_-S (2.2085 Å) is longer than that of W=S (2.1496 Å), but is shorter than those of W-µ_3_-S of (2.273 Å) and W-µ_5_-S (2.7102 Å). The Ag—S bond distances are in the range of 2.5140 (12)–2.8409 (12) Å. The average distance of Ag1(Ag2)—P (2.3919 Å) is shorter than that of Ag3(Ag4)—P.

Interestingly, the fragment [(WS_4_)_2_Ag_4_(dppm)_3_] in the title complex I is an isomer of [(WS_4_)_2_Ag_4_(dppm)_3_](II)(Yu *et al.*, 2001) (Figure 2). The main difference being the occurence of the µ_5_-S in complex I. Consequently, in complex I each Ag atom is four-coordinated, while in complex II two Ag atoms are three-coordinated, the other two Ag atoms are four-coordinated. The mean distance of W—S(2.086 Å) in I is shorter than that observed in complex II (2.136 Å). The average W—Ag length in complex I (3.0356 Å) is similar to that in complex II (3.0406 Å).

The formation of this two isomers may be related to the choice of the solvent. Complex I was synthesized in the mixed solvents DMF and MeCN, while complex II is obtained in CH_2_Cl_2_. In this paper, complex I was prepared by using different Ag salts as starting materials: AgBF_4_ or AgNO_3_. From this we know, the fragment [(WS_4_)_2_Ag_4_(dppm)_3_] may be easily obtained because of its stable structure with high symmetry.

### Experimental

The title complex was prepared by the reaction of AgNO_3_, bis(diphenylphosphino)methane(dppm), (NH_4_)_2_WS_4_ and Phen (phenathroline) in molar ratio of 2:4:1:0.5 in the mixed solvents MeCN and DMF(10 ml,V/V=1/1). The mixture was stirred at room temperature for 8 h, then filtered. Subsequent slow evaporation of the filtrate resulted in the formation of yellow crystals of the title complex. Crystals suitable for single-crystal X-ray diffraction were selected directly from the sample as prepared. Analysis found(percentage): C 41.33, H 3.40, N 1.18; calculated: C 41.31, H 3.42, N 1.19.

Alternative method to synthesize complex 1 is using AgBF_4_ in place of AgNO_3_ in the starting materials.

### Refinement

Metal atom centers were located from the E-maps and other non-hydrogen atoms were located in successive difference Fourier syntheses. The final refinements were performed by full matrix least-squares methods with anisotropic thermal parameters for non-hydrogen atoms on F^2^.

The final refinements were performed with isotropic thermal parameters. All hydrogen atoms were located in the calculated sites and included in the final refinement in the riding model approximation with displacement parameters derived from the parent atoms to which they were bonded.

### Crystal data

**Table:**

[Ag_4_W_2_S_8_(C_25_H_22_P_2_)_3_]·2C_3_H_7_NO	*F*(000) = 4600
*M**_r_* = 2354.95	*D*_x_ = 1.826 Mg m^−^^3^
Monoclinic, *P*2_1_/*c*	Mo *K*α radiation, λ = 0.71073 Å
Hall symbol: -P 2ybc	Cell parameters from 27248 reflections
*a* = 22.931 (2) Å	θ = 3.0–27.5°
*b* = 14.0395 (12) Å	µ = 3.93 mm^−^^1^
*c* = 27.855 (3) Å	*T* = 93 K
β = 107.224 (1)°	Block, yellow
*V* = 8565.6 (14) Å^3^	0.47 × 0.43 × 0.43 mm
*Z* = 4	

### Data collection

**Table:**

Rigaku R-AXIS RAPID diffractometer	19520 independent reflections
Radiation source: Rotating Anode	18777 reflections with *I* > 2σ(*I*)
graphite	*R*_int_ = 0.045
ω scans	θ_max_ = 27.5°, θ_min_ = 3.0°
Absorption correction: multi-scan (*ABSCOR*; Higashi, 1995)	*h* = −29→20
*T*_min_ = 0.262, *T*_max_ = 0.281	*k* = −18→18
69256 measured reflections	*l* = −35→36

### Refinement

**Table:**

Refinement on *F*^2^	Primary atom site location: structure-invariant direct methods
Least-squares matrix: full	Secondary atom site location: difference Fourier map
*R*[*F*^2^ > 2σ(*F*^2^)] = 0.039	Hydrogen site location: inferred from neighbouring sites
*wR*(*F*^2^) = 0.089	H-atom parameters constrained
*S* = 1.14	*w* = 1/[σ^2^(*F*_o_^2^) + (0.0341*P*)^2^ + 13.8363*P*] where *P* = (*F*_o_^2^ + 2*F*_c_^2^)/3
19520 reflections	(Δ/σ)_max_ = 0.009
950 parameters	Δρ_max_ = 1.20 e Å^−^^3^
0 restraints	Δρ_min_ = −1.36 e Å^−^^3^

### Special details

**Table:**

Geometry. All esds (except the esd in the dihedral angle between two l.s. planes) are estimated using the full covariance matrix. The cell esds are taken into account individually in the estimation of esds in distances, angles and torsion angles; correlations between esds in cell parameters are only used when they are defined by crystal symmetry. An approximate (isotropic) treatment of cell esds is used for estimating esds involving l.s. planes.
Refinement. Refinement of *F*^2^ against ALL reflections. The weighted *R*-factor *wR* and goodness of fit *S* are based on *F*^2^, conventional *R*-factors *R* are based on *F*, with *F* set to zero for negative *F*^2^. The threshold expression of *F*^2^ > σ(*F*^2^) is used only for calculating *R*-factors(gt) *etc*. and is not relevant to the choice of reflections for refinement. *R*-factors based on *F*^2^ are statistically about twice as large as those based on *F*, and *R*- factors based on ALL data will be even larger.

### Fractional atomic coordinates and isotropic or equivalent isotropic displacement parameters (Å^2^)

**Table:**

	*x*	*y*	*z*	*U*_iso_*/*U*_eq_	
W1	0.685156 (8)	0.527538 (12)	0.590934 (6)	0.00959 (5)	
W2	0.828154 (8)	0.329261 (13)	0.458854 (7)	0.01383 (5)	
Ag1	0.701283 (16)	0.55717 (2)	0.490031 (13)	0.01578 (7)	
Ag2	0.797387 (15)	0.41531 (2)	0.598751 (13)	0.01418 (7)	
Ag3	0.776781 (15)	0.20725 (2)	0.532723 (12)	0.01373 (7)	
Ag4	0.693052 (15)	0.34050 (2)	0.401396 (12)	0.01168 (7)	
S1	0.60897 (5)	0.52790 (8)	0.52055 (4)	0.0157 (2)	
S2	0.77015 (5)	0.59208 (8)	0.57931 (4)	0.0129 (2)	
S3	0.70064 (5)	0.37936 (8)	0.62017 (4)	0.0136 (2)	
S4	0.65859 (5)	0.61359 (8)	0.64489 (4)	0.0154 (2)	
S5	0.75460 (5)	0.37745 (8)	0.49394 (4)	0.0136 (2)	
S6	0.84065 (6)	0.17432 (9)	0.46535 (5)	0.0247 (3)	
S7	0.79958 (5)	0.36970 (9)	0.37903 (4)	0.0156 (2)	
S8	0.91157 (6)	0.40177 (12)	0.49661 (5)	0.0332 (3)	
P1	0.70165 (5)	0.64377 (8)	0.41615 (4)	0.0122 (2)	
P2	0.90214 (5)	0.37198 (8)	0.63471 (4)	0.0119 (2)	
P3	0.86722 (5)	0.16721 (8)	0.60311 (4)	0.0123 (2)	
P4	0.68282 (5)	0.11687 (8)	0.49608 (4)	0.0110 (2)	
P5	0.65584 (5)	0.17597 (8)	0.38414 (4)	0.0100 (2)	
P6	0.64487 (5)	0.47428 (8)	0.34787 (4)	0.0112 (2)	
C1	0.7730 (2)	0.7088 (3)	0.42479 (17)	0.0187 (10)	
C2	0.8271 (2)	0.6582 (4)	0.4461 (2)	0.0272 (12)	
H2	0.8255	0.5942	0.4567	0.033*	
C3	0.8832 (3)	0.7019 (5)	0.4518 (2)	0.0370 (15)	
H3	0.9200	0.6673	0.4655	0.044*	
C4	0.8853 (3)	0.7961 (5)	0.4374 (2)	0.0437 (18)	
H4	0.9236	0.8258	0.4412	0.052*	
C5	0.8326 (3)	0.8468 (5)	0.4178 (2)	0.0433 (17)	
H5	0.8345	0.9118	0.4090	0.052*	
C6	0.7761 (3)	0.8030 (4)	0.4107 (2)	0.0288 (12)	
H6	0.7396	0.8377	0.3962	0.035*	
C7	0.6402 (2)	0.7312 (3)	0.39761 (17)	0.0153 (9)	
C8	0.6100 (2)	0.7549 (3)	0.43287 (19)	0.0203 (10)	
H8	0.6220	0.7256	0.4651	0.024*	
C9	0.5629 (3)	0.8202 (4)	0.4214 (2)	0.0282 (12)	
H9	0.5431	0.8370	0.4457	0.034*	
C10	0.5449 (2)	0.8609 (4)	0.3743 (2)	0.0281 (12)	
H10	0.5121	0.9051	0.3661	0.034*	
C11	0.5742 (3)	0.8380 (4)	0.3387 (2)	0.0257 (11)	
H11	0.5612	0.8665	0.3064	0.031*	
C12	0.6221 (2)	0.7741 (3)	0.35032 (18)	0.0191 (10)	
H12	0.6426	0.7594	0.3262	0.023*	
C13	0.6964 (2)	0.5780 (3)	0.35824 (16)	0.0130 (9)	
H13A	0.7378	0.5560	0.3592	0.016*	
H13B	0.6820	0.6220	0.3293	0.016*	
C14	0.5696 (2)	0.5181 (3)	0.34616 (17)	0.0141 (9)	
C15	0.5491 (2)	0.5052 (3)	0.38825 (17)	0.0155 (9)	
H15	0.5748	0.4752	0.4174	0.019*	
C16	0.4916 (2)	0.5358 (3)	0.38777 (19)	0.0201 (10)	
H16	0.4786	0.5278	0.4168	0.024*	
C17	0.4532 (2)	0.5778 (3)	0.3455 (2)	0.0224 (11)	
H17	0.4136	0.5978	0.3453	0.027*	
C18	0.4728 (2)	0.5907 (4)	0.30313 (19)	0.0236 (11)	
H18	0.4464	0.6193	0.2738	0.028*	
C19	0.5311 (2)	0.5619 (3)	0.30380 (19)	0.0196 (10)	
H19	0.5446	0.5722	0.2751	0.024*	
C20	0.6363 (2)	0.4418 (3)	0.28259 (18)	0.0152 (9)	
C21	0.6730 (2)	0.4792 (3)	0.25509 (17)	0.0169 (9)	
H21	0.7017	0.5278	0.2694	0.020*	
C22	0.6680 (2)	0.4458 (4)	0.20679 (19)	0.0234 (11)	
H22	0.6928	0.4719	0.1882	0.028*	
C23	0.6265 (2)	0.3743 (4)	0.18612 (19)	0.0256 (11)	
H23	0.6230	0.3515	0.1533	0.031*	
C24	0.5905 (3)	0.3362 (4)	0.21297 (19)	0.0269 (12)	
H24	0.5630	0.2860	0.1989	0.032*	
C25	0.5941 (2)	0.3709 (3)	0.26080 (18)	0.0201 (10)	
H25	0.5678	0.3462	0.2785	0.024*	
C26	0.57227 (19)	0.1766 (3)	0.36167 (16)	0.0112 (8)	
C27	0.5416 (2)	0.2602 (3)	0.36530 (18)	0.0173 (9)	
H27	0.5644	0.3144	0.3807	0.021*	
C28	0.4787 (2)	0.2666 (4)	0.3470 (2)	0.0244 (11)	
H28	0.4587	0.3248	0.3496	0.029*	
C29	0.4448 (2)	0.1873 (4)	0.32495 (19)	0.0215 (10)	
H29	0.4016	0.1913	0.3118	0.026*	
C30	0.4751 (2)	0.1017 (3)	0.32236 (18)	0.0189 (10)	
H30	0.4522	0.0465	0.3087	0.023*	
C31	0.5378 (2)	0.0973 (3)	0.33959 (17)	0.0173 (9)	
H31	0.5580	0.0395	0.3364	0.021*	
C32	0.6776 (2)	0.1222 (3)	0.33224 (16)	0.0114 (8)	
C33	0.6938 (2)	0.1824 (3)	0.29875 (17)	0.0162 (9)	
H33	0.6965	0.2491	0.3049	0.019*	
C34	0.7063 (2)	0.1462 (4)	0.25613 (18)	0.0201 (10)	
H34	0.7169	0.1880	0.2333	0.024*	
C35	0.7029 (2)	0.0485 (4)	0.24736 (18)	0.0209 (10)	
H35	0.7113	0.0236	0.2184	0.025*	
C36	0.6874 (2)	−0.0127 (3)	0.28086 (18)	0.0200 (10)	
H36	0.6848	−0.0793	0.2747	0.024*	
C37	0.6757 (2)	0.0242 (3)	0.32364 (18)	0.0183 (10)	
H37	0.6664	−0.0178	0.3471	0.022*	
C38	0.6761 (2)	0.0792 (3)	0.43110 (16)	0.0134 (9)	
H38A	0.7154	0.0511	0.4304	0.016*	
H38B	0.6446	0.0288	0.4212	0.016*	
C39	0.6064 (2)	0.1611 (3)	0.49084 (16)	0.0119 (8)	
C40	0.5981 (2)	0.2566 (3)	0.50130 (18)	0.0187 (10)	
H40	0.6324	0.2970	0.5143	0.022*	
C41	0.5393 (2)	0.2920 (4)	0.4926 (2)	0.0233 (11)	
H41	0.5335	0.3568	0.4998	0.028*	
C42	0.4894 (2)	0.2340 (4)	0.4736 (2)	0.0248 (11)	
H42	0.4494	0.2594	0.4672	0.030*	
C43	0.4970 (2)	0.1386 (4)	0.46372 (18)	0.0193 (10)	
H43	0.4624	0.0985	0.4513	0.023*	
C44	0.5557 (2)	0.1020 (3)	0.47208 (17)	0.0159 (9)	
H44	0.5612	0.0370	0.4650	0.019*	
C45	0.6863 (2)	0.0014 (3)	0.52730 (17)	0.0144 (9)	
C46	0.7188 (2)	−0.0738 (3)	0.51465 (18)	0.0198 (10)	
H46	0.7371	−0.0662	0.4885	0.024*	
C47	0.7243 (3)	−0.1603 (4)	0.54060 (19)	0.0254 (11)	
H47	0.7460	−0.2117	0.5318	0.030*	
C48	0.6979 (2)	−0.1708 (4)	0.57924 (19)	0.0245 (11)	
H48	0.7016	−0.2295	0.5968	0.029*	
C49	0.6666 (2)	−0.0966 (4)	0.59207 (19)	0.0246 (11)	
H49	0.6486	−0.1044	0.6185	0.030*	
C50	0.6608 (2)	−0.0102 (4)	0.56679 (18)	0.0204 (10)	
H50	0.6395	0.0410	0.5763	0.024*	
C51	0.9045 (2)	0.0612 (3)	0.58746 (16)	0.0156 (9)	
C52	0.8728 (3)	−0.0241 (4)	0.5823 (2)	0.0297 (12)	
H52	0.8345	−0.0265	0.5890	0.036*	
C53	0.8965 (3)	−0.1062 (4)	0.5675 (2)	0.0377 (15)	
H53	0.8744	−0.1643	0.5638	0.045*	
C54	0.9519 (3)	−0.1034 (4)	0.5582 (2)	0.0327 (13)	
H54	0.9682	−0.1597	0.5483	0.039*	
C55	0.9841 (3)	−0.0190 (4)	0.5631 (2)	0.0297 (12)	
H55	1.0223	−0.0171	0.5563	0.036*	
C56	0.9603 (2)	0.0633 (4)	0.5781 (2)	0.0246 (11)	
H56	0.9826	0.1212	0.5819	0.030*	
C57	0.8633 (2)	0.1414 (3)	0.66613 (17)	0.0137 (9)	
C58	0.9155 (2)	0.1100 (3)	0.70346 (17)	0.0182 (10)	
H58	0.9518	0.0962	0.6950	0.022*	
C59	0.9142 (2)	0.0992 (4)	0.75240 (18)	0.0200 (10)	
H59	0.9496	0.0777	0.7775	0.024*	
C60	0.8614 (2)	0.1197 (4)	0.76500 (18)	0.0209 (10)	
H60	0.8610	0.1132	0.7989	0.025*	
C61	0.8097 (2)	0.1492 (3)	0.72898 (19)	0.0188 (10)	
H61	0.7737	0.1628	0.7379	0.023*	
C62	0.8100 (2)	0.1593 (3)	0.67889 (18)	0.0170 (9)	
H62	0.7739	0.1783	0.6538	0.020*	
C63	0.92682 (19)	0.2589 (3)	0.61403 (16)	0.0116 (8)	
H63A	0.9638	0.2360	0.6399	0.014*	
H63B	0.9377	0.2695	0.5826	0.014*	
C64	0.9232 (2)	0.3680 (3)	0.70338 (17)	0.0138 (9)	
C65	0.9833 (2)	0.3707 (4)	0.73330 (18)	0.0212 (10)	
H65	1.0153	0.3700	0.7181	0.025*	
C66	0.9974 (2)	0.3745 (4)	0.78542 (19)	0.0251 (11)	
H66	1.0388	0.3787	0.8055	0.030*	
C67	0.9514 (3)	0.3723 (4)	0.80778 (19)	0.0286 (12)	
H67	0.9612	0.3739	0.8434	0.034*	
C68	0.8912 (3)	0.3677 (4)	0.77874 (19)	0.0277 (12)	
H68	0.8595	0.3658	0.7943	0.033*	
C69	0.8771 (2)	0.3658 (3)	0.72647 (18)	0.0191 (10)	
H69	0.8357	0.3630	0.7065	0.023*	
C70	0.9557 (2)	0.4595 (3)	0.62351 (17)	0.0142 (9)	
C71	1.0132 (2)	0.4363 (3)	0.61976 (17)	0.0153 (9)	
H71	1.0264	0.3718	0.6227	0.018*	
C72	1.0514 (2)	0.5073 (3)	0.61178 (17)	0.0173 (9)	
H72	1.0904	0.4913	0.6087	0.021*	
C73	1.0329 (2)	0.6012 (4)	0.60827 (18)	0.0205 (10)	
H73	1.0593	0.6496	0.6030	0.025*	
C74	0.9756 (2)	0.6252 (4)	0.61239 (19)	0.0226 (11)	
H74	0.9633	0.6900	0.6108	0.027*	
C75	0.9368 (2)	0.5540 (3)	0.61882 (19)	0.0193 (10)	
H75	0.8970	0.5699	0.6200	0.023*	
O1	0.71217 (18)	0.7272 (3)	0.26992 (15)	0.0286 (9)	
N1	0.78968 (19)	0.6532 (3)	0.24912 (16)	0.0215 (9)	
C76	0.7377 (2)	0.7023 (3)	0.2391 (2)	0.0230 (11)	
H76	0.7188	0.7196	0.2050	0.028*	
C77	0.8220 (2)	0.6244 (4)	0.3001 (2)	0.0287 (12)	
H77A	0.8074	0.6621	0.3239	0.043*	
H77B	0.8659	0.6350	0.3065	0.043*	
H77C	0.8145	0.5567	0.3044	0.043*	
C78	0.8173 (3)	0.6276 (4)	0.2097 (2)	0.0303 (12)	
H78A	0.7921	0.6525	0.1773	0.045*	
H78B	0.8199	0.5581	0.2078	0.045*	
H78C	0.8583	0.6550	0.2177	0.045*	
O2	0.9365 (2)	0.6952 (4)	0.83007 (18)	0.0508 (13)	
N2	0.8745 (2)	0.6405 (3)	0.75669 (18)	0.0266 (10)	
C79	0.8866 (3)	0.6654 (4)	0.8036 (2)	0.0352 (14)	
H79	0.8546	0.6604	0.8186	0.042*	
C80	0.9225 (3)	0.6454 (5)	0.7310 (3)	0.0405 (15)	
H80A	0.9587	0.6772	0.7530	0.061*	
H80B	0.9072	0.6815	0.6997	0.061*	
H80C	0.9334	0.5808	0.7235	0.061*	
C81	0.8168 (3)	0.6078 (5)	0.7255 (3)	0.0424 (16)	
H81A	0.7874	0.6068	0.7448	0.064*	
H81B	0.8213	0.5434	0.7135	0.064*	
H81C	0.8021	0.6507	0.6966	0.064*	

### Atomic displacement parameters (Å^2^)

**Table:**

	*U*^11^	*U*^22^	*U*^33^	*U*^12^	*U*^13^	*U*^23^
W1	0.00959 (9)	0.01051 (8)	0.00897 (8)	0.00050 (6)	0.00322 (7)	0.00076 (6)
W2	0.01041 (9)	0.02124 (10)	0.00953 (9)	−0.00198 (7)	0.00248 (7)	0.00324 (7)
Ag1	0.01769 (18)	0.01876 (17)	0.01144 (16)	0.00004 (13)	0.00515 (13)	0.00419 (13)
Ag2	0.00979 (16)	0.01623 (16)	0.01571 (17)	0.00196 (12)	0.00253 (13)	0.00284 (13)
Ag3	0.01196 (17)	0.01387 (16)	0.01320 (16)	−0.00216 (12)	0.00038 (13)	0.00199 (12)
Ag4	0.01172 (16)	0.01111 (15)	0.01114 (15)	−0.00089 (12)	0.00175 (12)	0.00073 (12)
S1	0.0119 (5)	0.0230 (6)	0.0107 (5)	−0.0008 (4)	0.0009 (4)	0.0018 (4)
S2	0.0118 (5)	0.0124 (5)	0.0151 (5)	−0.0015 (4)	0.0049 (4)	0.0014 (4)
S3	0.0149 (5)	0.0121 (5)	0.0154 (5)	0.0014 (4)	0.0069 (4)	0.0030 (4)
S4	0.0166 (6)	0.0149 (5)	0.0165 (5)	0.0016 (4)	0.0077 (4)	−0.0022 (4)
S5	0.0178 (6)	0.0134 (5)	0.0100 (5)	−0.0002 (4)	0.0047 (4)	0.0002 (4)
S6	0.0313 (7)	0.0250 (6)	0.0236 (6)	0.0131 (5)	0.0168 (6)	0.0079 (5)
S7	0.0139 (6)	0.0241 (6)	0.0093 (5)	−0.0017 (4)	0.0043 (4)	0.0032 (4)
S8	0.0199 (7)	0.0608 (10)	0.0151 (6)	−0.0208 (6)	−0.0006 (5)	0.0056 (6)
P1	0.0146 (6)	0.0116 (5)	0.0102 (5)	−0.0019 (4)	0.0036 (4)	0.0007 (4)
P2	0.0103 (5)	0.0127 (5)	0.0118 (5)	−0.0002 (4)	0.0019 (4)	0.0012 (4)
P3	0.0126 (6)	0.0123 (5)	0.0105 (5)	−0.0008 (4)	0.0012 (4)	0.0014 (4)
P4	0.0119 (5)	0.0107 (5)	0.0099 (5)	−0.0015 (4)	0.0026 (4)	0.0009 (4)
P5	0.0110 (5)	0.0100 (5)	0.0089 (5)	−0.0003 (4)	0.0027 (4)	0.0002 (4)
P6	0.0111 (6)	0.0112 (5)	0.0111 (5)	0.0000 (4)	0.0032 (4)	0.0017 (4)
C1	0.024 (3)	0.021 (2)	0.014 (2)	−0.0047 (19)	0.0099 (19)	−0.0057 (19)
C2	0.020 (3)	0.031 (3)	0.030 (3)	−0.004 (2)	0.006 (2)	−0.009 (2)
C3	0.021 (3)	0.056 (4)	0.034 (3)	−0.010 (3)	0.007 (2)	−0.016 (3)
C4	0.039 (4)	0.067 (5)	0.032 (3)	−0.035 (3)	0.020 (3)	−0.021 (3)
C5	0.050 (4)	0.041 (4)	0.040 (4)	−0.030 (3)	0.016 (3)	−0.005 (3)
C6	0.034 (3)	0.027 (3)	0.025 (3)	−0.015 (2)	0.009 (2)	0.003 (2)
C7	0.018 (2)	0.011 (2)	0.017 (2)	−0.0027 (17)	0.0049 (19)	0.0010 (17)
C8	0.025 (3)	0.016 (2)	0.021 (2)	−0.0004 (19)	0.010 (2)	−0.0008 (19)
C9	0.032 (3)	0.022 (3)	0.036 (3)	0.004 (2)	0.019 (3)	0.004 (2)
C10	0.024 (3)	0.022 (3)	0.039 (3)	0.004 (2)	0.009 (2)	0.005 (2)
C11	0.029 (3)	0.019 (2)	0.029 (3)	0.002 (2)	0.008 (2)	0.006 (2)
C12	0.028 (3)	0.015 (2)	0.015 (2)	0.0009 (19)	0.008 (2)	0.0018 (18)
C13	0.014 (2)	0.014 (2)	0.010 (2)	−0.0012 (17)	0.0027 (17)	0.0031 (17)
C14	0.015 (2)	0.014 (2)	0.015 (2)	−0.0031 (17)	0.0063 (18)	−0.0027 (17)
C15	0.015 (2)	0.015 (2)	0.016 (2)	−0.0023 (17)	0.0044 (18)	−0.0044 (18)
C16	0.018 (3)	0.024 (3)	0.019 (2)	−0.0012 (19)	0.008 (2)	−0.003 (2)
C17	0.015 (2)	0.020 (2)	0.034 (3)	0.0037 (19)	0.009 (2)	−0.001 (2)
C18	0.020 (3)	0.026 (3)	0.021 (3)	0.004 (2)	0.000 (2)	0.004 (2)
C19	0.019 (3)	0.021 (2)	0.021 (2)	0.0055 (19)	0.008 (2)	0.006 (2)
C20	0.014 (2)	0.010 (2)	0.020 (2)	0.0033 (17)	0.0036 (18)	0.0031 (18)
C21	0.013 (2)	0.022 (2)	0.015 (2)	0.0022 (18)	0.0031 (18)	0.0002 (18)
C22	0.023 (3)	0.032 (3)	0.016 (2)	0.009 (2)	0.007 (2)	0.001 (2)
C23	0.031 (3)	0.028 (3)	0.014 (2)	0.003 (2)	0.001 (2)	−0.003 (2)
C24	0.042 (3)	0.015 (2)	0.017 (2)	−0.005 (2)	−0.002 (2)	−0.0003 (19)
C25	0.025 (3)	0.016 (2)	0.016 (2)	−0.0064 (19)	0.000 (2)	0.0033 (18)
C26	0.009 (2)	0.017 (2)	0.0078 (19)	0.0001 (16)	0.0029 (16)	0.0040 (16)
C27	0.018 (2)	0.016 (2)	0.019 (2)	−0.0002 (18)	0.0084 (19)	0.0023 (18)
C28	0.019 (3)	0.020 (2)	0.037 (3)	0.005 (2)	0.012 (2)	0.000 (2)
C29	0.011 (2)	0.033 (3)	0.022 (3)	−0.002 (2)	0.0048 (19)	0.002 (2)
C30	0.018 (2)	0.021 (2)	0.017 (2)	−0.0063 (19)	0.0049 (19)	−0.0068 (19)
C31	0.019 (2)	0.018 (2)	0.014 (2)	−0.0008 (18)	0.0030 (19)	−0.0003 (18)
C32	0.013 (2)	0.012 (2)	0.0088 (19)	0.0016 (16)	0.0031 (16)	−0.0014 (16)
C33	0.018 (2)	0.014 (2)	0.017 (2)	0.0010 (17)	0.0056 (19)	0.0008 (18)
C34	0.020 (3)	0.026 (3)	0.017 (2)	−0.003 (2)	0.010 (2)	0.002 (2)
C35	0.024 (3)	0.027 (3)	0.013 (2)	0.000 (2)	0.007 (2)	−0.004 (2)
C36	0.028 (3)	0.011 (2)	0.020 (2)	0.0032 (19)	0.006 (2)	−0.0052 (18)
C37	0.025 (3)	0.017 (2)	0.014 (2)	0.0002 (19)	0.007 (2)	0.0025 (18)
C38	0.016 (2)	0.012 (2)	0.012 (2)	−0.0024 (17)	0.0048 (18)	−0.0011 (17)
C39	0.011 (2)	0.017 (2)	0.0081 (19)	−0.0030 (16)	0.0038 (16)	0.0013 (16)
C40	0.018 (2)	0.017 (2)	0.019 (2)	−0.0022 (18)	0.0034 (19)	−0.0039 (19)
C41	0.023 (3)	0.017 (2)	0.033 (3)	0.003 (2)	0.013 (2)	−0.002 (2)
C42	0.018 (3)	0.033 (3)	0.025 (3)	0.003 (2)	0.009 (2)	−0.003 (2)
C43	0.015 (2)	0.023 (2)	0.019 (2)	−0.0064 (19)	0.0053 (19)	−0.005 (2)
C44	0.017 (2)	0.017 (2)	0.015 (2)	−0.0040 (18)	0.0071 (18)	−0.0027 (18)
C45	0.013 (2)	0.018 (2)	0.012 (2)	−0.0051 (17)	0.0032 (17)	0.0018 (17)
C46	0.028 (3)	0.017 (2)	0.015 (2)	−0.0001 (19)	0.006 (2)	0.0006 (18)
C47	0.037 (3)	0.017 (2)	0.018 (2)	0.000 (2)	0.001 (2)	−0.001 (2)
C48	0.030 (3)	0.018 (2)	0.019 (3)	−0.006 (2)	−0.003 (2)	0.002 (2)
C49	0.027 (3)	0.030 (3)	0.017 (2)	−0.006 (2)	0.007 (2)	0.009 (2)
C50	0.024 (3)	0.024 (2)	0.015 (2)	0.001 (2)	0.007 (2)	0.0054 (19)
C51	0.019 (2)	0.014 (2)	0.010 (2)	0.0035 (18)	−0.0017 (18)	0.0002 (17)
C52	0.028 (3)	0.023 (3)	0.037 (3)	0.000 (2)	0.008 (3)	−0.003 (2)
C53	0.044 (4)	0.015 (3)	0.051 (4)	−0.004 (2)	0.008 (3)	−0.012 (3)
C54	0.050 (4)	0.024 (3)	0.020 (3)	0.011 (3)	0.003 (3)	−0.008 (2)
C55	0.034 (3)	0.027 (3)	0.029 (3)	0.013 (2)	0.011 (2)	0.004 (2)
C56	0.027 (3)	0.020 (2)	0.029 (3)	0.004 (2)	0.011 (2)	0.003 (2)
C57	0.014 (2)	0.013 (2)	0.013 (2)	−0.0018 (17)	0.0026 (17)	0.0019 (17)
C58	0.015 (2)	0.026 (2)	0.014 (2)	−0.0007 (19)	0.0050 (18)	0.0014 (19)
C59	0.019 (3)	0.025 (3)	0.013 (2)	0.001 (2)	0.0019 (19)	0.0066 (19)
C60	0.026 (3)	0.024 (2)	0.014 (2)	−0.002 (2)	0.009 (2)	0.0015 (19)
C61	0.018 (2)	0.018 (2)	0.024 (3)	−0.0007 (18)	0.013 (2)	0.0030 (19)
C62	0.014 (2)	0.015 (2)	0.022 (2)	−0.0009 (17)	0.0066 (19)	0.0015 (18)
C63	0.008 (2)	0.016 (2)	0.012 (2)	−0.0002 (16)	0.0050 (16)	0.0009 (17)
C64	0.017 (2)	0.0093 (19)	0.013 (2)	−0.0019 (17)	0.0017 (18)	−0.0012 (16)
C65	0.018 (3)	0.027 (3)	0.016 (2)	0.002 (2)	0.0018 (19)	0.001 (2)
C66	0.020 (3)	0.030 (3)	0.019 (2)	−0.002 (2)	−0.005 (2)	−0.001 (2)
C67	0.035 (3)	0.036 (3)	0.011 (2)	−0.002 (2)	0.001 (2)	−0.005 (2)
C68	0.034 (3)	0.036 (3)	0.017 (3)	−0.005 (2)	0.013 (2)	−0.006 (2)
C69	0.018 (2)	0.021 (2)	0.018 (2)	−0.0015 (19)	0.0045 (19)	−0.0052 (19)
C70	0.012 (2)	0.017 (2)	0.012 (2)	−0.0031 (17)	0.0015 (17)	0.0004 (17)
C71	0.013 (2)	0.017 (2)	0.014 (2)	−0.0020 (17)	0.0021 (18)	0.0007 (18)
C72	0.016 (2)	0.024 (2)	0.015 (2)	−0.0050 (19)	0.0088 (19)	−0.0020 (19)
C73	0.021 (3)	0.023 (2)	0.016 (2)	−0.008 (2)	0.004 (2)	0.0018 (19)
C74	0.021 (3)	0.017 (2)	0.027 (3)	−0.0029 (19)	0.004 (2)	0.004 (2)
C75	0.010 (2)	0.018 (2)	0.028 (3)	−0.0020 (18)	0.0035 (19)	−0.001 (2)
O1	0.031 (2)	0.0237 (19)	0.035 (2)	0.0058 (16)	0.0170 (18)	0.0087 (17)
N1	0.020 (2)	0.022 (2)	0.023 (2)	−0.0003 (17)	0.0074 (18)	0.0044 (18)
C76	0.021 (3)	0.017 (2)	0.031 (3)	0.0007 (19)	0.009 (2)	0.012 (2)
C77	0.022 (3)	0.034 (3)	0.026 (3)	−0.001 (2)	0.001 (2)	0.008 (2)
C78	0.029 (3)	0.028 (3)	0.037 (3)	0.006 (2)	0.014 (3)	0.004 (2)
O2	0.026 (2)	0.071 (3)	0.043 (3)	−0.006 (2)	−0.008 (2)	−0.016 (3)
N2	0.017 (2)	0.028 (2)	0.032 (3)	0.0030 (18)	0.0038 (19)	−0.007 (2)
C79	0.029 (3)	0.043 (4)	0.032 (3)	0.004 (3)	0.005 (3)	−0.001 (3)
C80	0.039 (4)	0.045 (4)	0.043 (4)	0.001 (3)	0.020 (3)	−0.006 (3)
C81	0.027 (3)	0.034 (3)	0.056 (4)	−0.002 (3)	−0.003 (3)	−0.016 (3)

### Geometric parameters (Å, °)

**Table:**

W1—S4	2.1523 (11)	C32—C33	1.390 (6)
W1—S1	2.2062 (11)	C32—C37	1.395 (6)
W1—S3	2.2240 (11)	C33—C34	1.396 (6)
W1—S2	2.2579 (11)	C33—H33	0.9500
W1—Ag2	2.9701 (4)	C34—C35	1.391 (7)
W1—Ag1	2.9707 (5)	C34—H34	0.9500
W2—S8	2.1470 (13)	C35—C36	1.390 (7)
W2—S6	2.1945 (13)	C35—H35	0.9500
W2—S7	2.1985 (11)	C36—C37	1.396 (6)
W2—S5	2.2878 (11)	C36—H36	0.9500
W2—Ag4	3.0410 (4)	C37—H37	0.9500
W2—Ag3	3.1609 (4)	C38—H38A	0.9900
Ag1—P1	2.3924 (12)	C38—H38B	0.9900
Ag1—S1	2.5374 (12)	C39—C40	1.396 (6)
Ag1—S2	2.5677 (12)	C39—C44	1.398 (6)
Ag1—S5	2.7919 (12)	C40—C41	1.390 (7)
Ag2—P2	2.3914 (12)	C40—H40	0.9500
Ag2—S3	2.5132 (11)	C41—C42	1.376 (7)
Ag2—S2	2.5772 (11)	C41—H41	0.9500
Ag2—S5	2.8412 (12)	C42—C43	1.389 (7)
Ag3—P4	2.4478 (12)	C42—H42	0.9500
Ag3—P3	2.4602 (12)	C43—C44	1.394 (7)
Ag3—S5	2.6096 (12)	C43—H43	0.9500
Ag3—S6	2.7403 (13)	C44—H44	0.9500
Ag4—P6	2.4485 (12)	C45—C46	1.396 (7)
Ag4—P5	2.4601 (11)	C45—C50	1.400 (6)
Ag4—S5	2.5984 (12)	C46—C47	1.400 (7)
Ag4—S7	2.7248 (12)	C46—H46	0.9500
P1—C1	1.826 (5)	C47—C48	1.390 (8)
P1—C7	1.824 (5)	C47—H47	0.9500
P1—C13	1.831 (4)	C48—C49	1.371 (8)
P2—C70	1.829 (5)	C48—H48	0.9500
P2—C64	1.830 (5)	C49—C50	1.389 (7)
P2—C63	1.835 (4)	C49—H49	0.9500
P3—C57	1.820 (5)	C50—H50	0.9500
P3—C51	1.831 (5)	C51—C56	1.380 (7)
P3—C63	1.836 (4)	C51—C52	1.387 (7)
P4—C39	1.823 (5)	C52—C53	1.390 (8)
P4—C45	1.830 (5)	C52—H52	0.9500
P4—C38	1.848 (4)	C53—C54	1.369 (9)
P5—C32	1.826 (4)	C53—H53	0.9500
P5—C26	1.832 (4)	C54—C55	1.381 (8)
P5—C38	1.847 (4)	C54—H54	0.9500
P6—C14	1.820 (5)	C55—C56	1.394 (7)
P6—C20	1.828 (5)	C55—H55	0.9500
P6—C13	1.844 (4)	C56—H56	0.9500
C1—C6	1.387 (7)	C57—C62	1.395 (6)
C1—C2	1.399 (7)	C57—C58	1.403 (6)
C2—C3	1.392 (8)	C58—C59	1.381 (6)
C2—H2	0.9500	C58—H58	0.9500
C3—C4	1.386 (10)	C59—C60	1.387 (7)
C3—H3	0.9500	C59—H59	0.9500
C4—C5	1.369 (10)	C60—C61	1.371 (7)
C4—H4	0.9500	C60—H60	0.9500
C5—C6	1.394 (8)	C61—C62	1.404 (7)
C5—H5	0.9500	C61—H61	0.9500
C6—H6	0.9500	C62—H62	0.9500
C7—C12	1.395 (6)	C63—H63A	0.9900
C7—C8	1.400 (6)	C63—H63B	0.9900
C8—C9	1.380 (7)	C64—C69	1.389 (6)
C8—H8	0.9500	C64—C65	1.385 (7)
C9—C10	1.376 (8)	C65—C66	1.392 (7)
C9—H9	0.9500	C65—H65	0.9500
C10—C11	1.388 (8)	C66—C67	1.375 (8)
C10—H10	0.9500	C66—H66	0.9500
C11—C12	1.381 (7)	C67—C68	1.380 (8)
C11—H11	0.9500	C67—H67	0.9500
C12—H12	0.9500	C68—C69	1.395 (7)
C13—H13A	0.9900	C68—H68	0.9500
C13—H13B	0.9900	C69—H69	0.9500
C14—C19	1.390 (7)	C70—C71	1.392 (6)
C14—C15	1.399 (6)	C70—C75	1.390 (6)
C15—C16	1.381 (7)	C71—C72	1.388 (6)
C15—H15	0.9500	C71—H71	0.9500
C16—C17	1.376 (7)	C72—C73	1.378 (7)
C16—H16	0.9500	C72—H72	0.9500
C17—C18	1.394 (7)	C73—C74	1.394 (7)
C17—H17	0.9500	C73—H73	0.9500
C18—C19	1.390 (7)	C74—C75	1.385 (6)
C18—H18	0.9500	C74—H74	0.9500
C19—H19	0.9500	C75—H75	0.9500
C20—C21	1.398 (6)	O1—C76	1.224 (6)
C20—C25	1.396 (6)	N1—C76	1.333 (6)
C21—C22	1.397 (6)	N1—C77	1.452 (6)
C21—H21	0.9500	N1—C78	1.465 (7)
C22—C23	1.384 (8)	C76—H76	0.9500
C22—H22	0.9500	C77—H77A	0.9800
C23—C24	1.376 (8)	C77—H77B	0.9800
C23—H23	0.9500	C77—H77C	0.9800
C24—C25	1.398 (7)	C78—H78A	0.9800
C24—H24	0.9500	C78—H78B	0.9800
C25—H25	0.9500	C78—H78C	0.9800
C26—C27	1.387 (6)	O2—C79	1.237 (7)
C26—C31	1.399 (6)	N2—C79	1.300 (7)
C27—C28	1.383 (7)	N2—C81	1.427 (7)
C27—H27	0.9500	N2—C80	1.481 (7)
C28—C29	1.392 (7)	C79—H79	0.9500
C28—H28	0.9500	C80—H80A	0.9800
C29—C30	1.401 (7)	C80—H80B	0.9800
C29—H29	0.9500	C80—H80C	0.9800
C30—C31	1.376 (7)	C81—H81A	0.9800
C30—H30	0.9500	C81—H81B	0.9800
C31—H31	0.9500	C81—H81C	0.9800
			
S4—W1—S1	107.81 (4)	C25—C24—H24	119.8
S4—W1—S3	108.47 (4)	C20—C25—C24	120.0 (5)
S1—W1—S3	109.03 (4)	C20—C25—H25	120.0
S4—W1—S2	108.04 (4)	C24—C25—H25	120.0
S1—W1—S2	111.20 (4)	C27—C26—C31	118.3 (4)
S3—W1—S2	112.14 (4)	C27—C26—P5	118.7 (3)
S4—W1—Ag2	131.67 (3)	C31—C26—P5	123.0 (3)
S1—W1—Ag2	120.49 (3)	C28—C27—C26	121.5 (5)
S3—W1—Ag2	55.67 (3)	C28—C27—H27	119.2
S2—W1—Ag2	57.14 (3)	C26—C27—H27	119.2
S4—W1—Ag1	136.25 (3)	C27—C28—C29	119.8 (5)
S1—W1—Ag1	56.40 (3)	C27—C28—H28	120.1
S3—W1—Ag1	115.24 (3)	C29—C28—H28	120.1
S2—W1—Ag1	56.88 (3)	C28—C29—C30	119.2 (5)
Ag2—W1—Ag1	78.214 (10)	C28—C29—H29	120.4
S8—W2—S6	110.73 (6)	C30—C29—H29	120.4
S8—W2—S7	109.18 (5)	C31—C30—C29	120.2 (4)
S6—W2—S7	109.38 (5)	C31—C30—H30	119.9
S8—W2—S5	108.25 (5)	C29—C30—H30	119.9
S6—W2—S5	110.66 (4)	C30—C31—C26	120.9 (4)
S7—W2—S5	108.61 (4)	C30—C31—H31	119.5
S8—W2—Ag4	148.67 (5)	C26—C31—H31	119.5
S6—W2—Ag4	100.53 (4)	C33—C32—C37	119.0 (4)
S7—W2—Ag4	60.14 (3)	C33—C32—P5	118.1 (3)
S5—W2—Ag4	56.25 (3)	C37—C32—P5	122.8 (3)
S8—W2—Ag3	112.82 (4)	C32—C33—C34	120.8 (4)
S6—W2—Ag3	58.26 (3)	C32—C33—H33	119.6
S7—W2—Ag3	137.85 (3)	C34—C33—H33	119.6
S5—W2—Ag3	54.42 (3)	C33—C34—C35	119.6 (4)
Ag4—W2—Ag3	81.533 (11)	C33—C34—H34	120.2
P1—Ag1—S1	125.90 (4)	C35—C34—H34	120.2
P1—Ag1—S2	125.34 (4)	C36—C35—C34	120.2 (4)
S1—Ag1—S2	92.36 (4)	C36—C35—H35	119.9
P1—Ag1—S5	112.32 (4)	C34—C35—H35	119.9
S1—Ag1—S5	103.87 (4)	C35—C36—C37	119.7 (4)
S2—Ag1—S5	89.89 (3)	C35—C36—H36	120.2
P1—Ag1—W1	156.66 (3)	C37—C36—H36	120.2
S1—Ag1—W1	46.40 (3)	C32—C37—C36	120.6 (4)
S2—Ag1—W1	47.43 (2)	C32—C37—H37	119.7
S5—Ag1—W1	90.70 (2)	C36—C37—H37	119.7
P2—Ag2—S3	134.21 (4)	P5—C38—P4	114.5 (2)
P2—Ag2—S2	118.94 (4)	P5—C38—H38A	108.6
S3—Ag2—S2	93.86 (4)	P4—C38—H38A	108.6
P2—Ag2—S5	112.41 (4)	P5—C38—H38B	108.6
S3—Ag2—S5	98.39 (4)	P4—C38—H38B	108.6
S2—Ag2—S5	88.61 (3)	H38A—C38—H38B	107.6
P2—Ag2—W1	155.06 (3)	C40—C39—C44	119.8 (4)
S3—Ag2—W1	46.95 (3)	C40—C39—P4	119.9 (3)
S2—Ag2—W1	47.38 (2)	C44—C39—P4	120.1 (3)
S5—Ag2—W1	89.76 (2)	C41—C40—C39	119.5 (4)
P4—Ag3—P3	130.09 (4)	C41—C40—H40	120.3
P4—Ag3—S5	105.22 (4)	C39—C40—H40	120.3
P3—Ag3—S5	123.65 (4)	C42—C41—C40	120.6 (5)
P4—Ag3—S6	102.12 (4)	C42—C41—H41	119.7
P3—Ag3—S6	90.68 (4)	C40—C41—H41	119.7
S5—Ag3—S6	87.08 (4)	C41—C42—C43	120.5 (5)
P4—Ag3—W2	117.46 (3)	C41—C42—H42	119.8
P3—Ag3—W2	104.71 (3)	C43—C42—H42	119.8
S5—Ag3—W2	45.48 (2)	C42—C43—C44	119.6 (4)
S6—Ag3—W2	42.93 (3)	C42—C43—H43	120.2
P6—Ag4—P5	122.24 (4)	C44—C43—H43	120.2
P6—Ag4—S5	117.74 (4)	C39—C44—C43	120.0 (4)
P5—Ag4—S5	115.87 (4)	C39—C44—H44	120.0
P6—Ag4—S7	91.08 (4)	C43—C44—H44	120.0
P5—Ag4—S7	112.28 (4)	C46—C45—C50	119.2 (4)
S5—Ag4—S7	86.36 (4)	C46—C45—P4	120.1 (3)
P6—Ag4—W2	124.47 (3)	C50—C45—P4	120.5 (4)
P5—Ag4—W2	107.12 (3)	C45—C46—C47	119.9 (5)
S5—Ag4—W2	47.06 (3)	C45—C46—H46	120.0
S7—Ag4—W2	44.41 (2)	C47—C46—H46	120.0
W1—S1—Ag1	77.20 (4)	C48—C47—C46	119.9 (5)
W1—S2—Ag1	75.69 (3)	C48—C47—H47	120.0
W1—S2—Ag2	75.48 (3)	C46—C47—H47	120.0
Ag1—S2—Ag2	93.50 (4)	C49—C48—C47	120.2 (5)
W1—S3—Ag2	77.39 (3)	C49—C48—H48	119.9
W2—S5—Ag4	76.69 (3)	C47—C48—H48	119.9
W2—S5—Ag3	80.10 (3)	C48—C49—C50	120.7 (5)
Ag4—S5—Ag3	102.12 (4)	C48—C49—H49	119.7
W2—S5—Ag1	128.34 (4)	C50—C49—H49	119.7
Ag4—S5—Ag1	92.22 (4)	C49—C50—C45	120.0 (5)
Ag3—S5—Ag1	150.82 (5)	C49—C50—H50	120.0
W2—S5—Ag2	114.86 (4)	C45—C50—H50	120.0
Ag4—S5—Ag2	167.86 (5)	C56—C51—C52	119.1 (5)
Ag3—S5—Ag2	77.31 (3)	C56—C51—P3	123.5 (4)
Ag1—S5—Ag2	83.40 (3)	C52—C51—P3	117.4 (4)
W2—S6—Ag3	78.81 (4)	C51—C52—C53	120.6 (6)
W2—S7—Ag4	75.45 (3)	C51—C52—H52	119.7
C1—P1—C7	106.7 (2)	C53—C52—H52	119.7
C1—P1—C13	101.0 (2)	C54—C53—C52	119.9 (5)
C7—P1—C13	104.3 (2)	C54—C53—H53	120.0
C1—P1—Ag1	111.83 (16)	C52—C53—H53	120.0
C7—P1—Ag1	112.73 (15)	C53—C54—C55	120.3 (5)
C13—P1—Ag1	119.05 (14)	C53—C54—H54	119.8
C70—P2—C64	102.5 (2)	C55—C54—H54	119.8
C70—P2—C63	104.0 (2)	C56—C55—C54	119.7 (6)
C64—P2—C63	106.9 (2)	C56—C55—H55	120.1
C70—P2—Ag2	113.57 (15)	C54—C55—H55	120.1
C64—P2—Ag2	111.52 (15)	C51—C56—C55	120.4 (5)
C63—P2—Ag2	116.97 (15)	C51—C56—H56	119.8
C57—P3—C51	103.1 (2)	C55—C56—H56	119.8
C57—P3—C63	103.1 (2)	C62—C57—C58	119.2 (4)
C51—P3—C63	103.4 (2)	C62—C57—P3	120.5 (3)
C57—P3—Ag3	122.93 (15)	C58—C57—P3	120.2 (3)
C51—P3—Ag3	109.79 (15)	C59—C58—C57	120.2 (4)
C63—P3—Ag3	112.48 (15)	C59—C58—H58	119.9
C39—P4—C45	104.2 (2)	C57—C58—H58	119.9
C39—P4—C38	102.7 (2)	C58—C59—C60	120.2 (5)
C45—P4—C38	101.0 (2)	C58—C59—H59	119.9
C39—P4—Ag3	124.25 (15)	C60—C59—H59	119.9
C45—P4—Ag3	110.63 (15)	C61—C60—C59	120.5 (4)
C38—P4—Ag3	111.39 (15)	C61—C60—H60	119.7
C32—P5—C26	103.6 (2)	C59—C60—H60	119.7
C32—P5—C38	100.7 (2)	C60—C61—C62	120.1 (4)
C26—P5—C38	105.3 (2)	C60—C61—H61	120.0
C32—P5—Ag4	112.51 (15)	C62—C61—H61	120.0
C26—P5—Ag4	109.23 (15)	C57—C62—C61	119.8 (4)
C38—P5—Ag4	123.52 (15)	C57—C62—H62	120.1
C14—P6—C20	103.7 (2)	C61—C62—H62	120.1
C14—P6—C13	107.2 (2)	P2—C63—P3	112.0 (2)
C20—P6—C13	103.2 (2)	P2—C63—H63A	109.2
C14—P6—Ag4	121.93 (15)	P3—C63—H63A	109.2
C20—P6—Ag4	108.08 (14)	P2—C63—H63B	109.2
C13—P6—Ag4	110.97 (15)	P3—C63—H63B	109.2
C6—C1—C2	119.4 (5)	H63A—C63—H63B	107.9
C6—C1—P1	123.9 (4)	C69—C64—C65	118.7 (4)
C2—C1—P1	116.7 (4)	C69—C64—P2	118.9 (4)
C3—C2—C1	119.9 (6)	C65—C64—P2	122.4 (4)
C3—C2—H2	120.1	C64—C65—C66	120.7 (5)
C1—C2—H2	120.1	C64—C65—H65	119.6
C2—C3—C4	119.8 (6)	C66—C65—H65	119.6
C2—C3—H3	120.1	C67—C66—C65	120.0 (5)
C4—C3—H3	120.1	C67—C66—H66	120.0
C5—C4—C3	120.6 (6)	C65—C66—H66	120.0
C5—C4—H4	119.7	C66—C67—C68	120.3 (5)
C3—C4—H4	119.7	C66—C67—H67	119.9
C4—C5—C6	120.1 (6)	C68—C67—H67	119.9
C4—C5—H5	120.0	C67—C68—C69	119.6 (5)
C6—C5—H5	120.0	C67—C68—H68	120.2
C1—C6—C5	120.2 (6)	C69—C68—H68	120.2
C1—C6—H6	119.9	C64—C69—C68	120.7 (5)
C5—C6—H6	119.9	C64—C69—H69	119.7
C12—C7—C8	119.3 (4)	C68—C69—H69	119.7
C12—C7—P1	123.2 (4)	C71—C70—C75	119.5 (4)
C8—C7—P1	117.6 (4)	C71—C70—P2	123.7 (4)
C9—C8—C7	120.7 (5)	C75—C70—P2	116.8 (3)
C9—C8—H8	119.6	C70—C71—C72	120.0 (4)
C7—C8—H8	119.6	C70—C71—H71	120.0
C8—C9—C10	119.4 (5)	C72—C71—H71	120.0
C8—C9—H9	120.3	C73—C72—C71	120.2 (4)
C10—C9—H9	120.3	C73—C72—H72	119.9
C9—C10—C11	120.8 (5)	C71—C72—H72	119.9
C9—C10—H10	119.6	C72—C73—C74	120.2 (4)
C11—C10—H10	119.6	C72—C73—H73	119.9
C12—C11—C10	120.2 (5)	C74—C73—H73	119.9
C12—C11—H11	119.9	C75—C74—C73	119.6 (5)
C10—C11—H11	119.9	C75—C74—H74	120.2
C11—C12—C7	119.7 (5)	C73—C74—H74	120.2
C11—C12—H12	120.2	C74—C75—C70	120.4 (5)
C7—C12—H12	120.2	C74—C75—H75	119.8
P1—C13—P6	114.1 (2)	C70—C75—H75	119.8
P1—C13—H13A	108.7	C76—N1—C77	121.0 (5)
P6—C13—H13A	108.7	C76—N1—C78	121.9 (4)
P1—C13—H13B	108.7	C77—N1—C78	117.1 (4)
P6—C13—H13B	108.7	O1—C76—N1	125.7 (5)
H13A—C13—H13B	107.6	O1—C76—H76	117.1
C19—C14—C15	118.8 (4)	N1—C76—H76	117.1
C19—C14—P6	122.1 (3)	N1—C77—H77A	109.5
C15—C14—P6	119.1 (4)	N1—C77—H77B	109.5
C16—C15—C14	120.5 (4)	H77A—C77—H77B	109.5
C16—C15—H15	119.7	N1—C77—H77C	109.5
C14—C15—H15	119.7	H77A—C77—H77C	109.5
C17—C16—C15	120.6 (5)	H77B—C77—H77C	109.5
C17—C16—H16	119.7	N1—C78—H78A	109.5
C15—C16—H16	119.7	N1—C78—H78B	109.5
C16—C17—C18	119.7 (5)	H78A—C78—H78B	109.5
C16—C17—H17	120.2	N1—C78—H78C	109.5
C18—C17—H17	120.2	H78A—C78—H78C	109.5
C19—C18—C17	120.0 (5)	H78B—C78—H78C	109.5
C19—C18—H18	120.0	C79—N2—C81	125.7 (5)
C17—C18—H18	120.0	C79—N2—C80	120.2 (5)
C14—C19—C18	120.4 (4)	C81—N2—C80	114.1 (5)
C14—C19—H19	119.8	O2—C79—N2	125.1 (6)
C18—C19—H19	119.8	O2—C79—H79	117.4
C21—C20—C25	118.8 (4)	N2—C79—H79	117.4
C21—C20—P6	123.1 (4)	N2—C80—H80A	109.5
C25—C20—P6	117.8 (4)	N2—C80—H80B	109.5
C22—C21—C20	120.7 (5)	H80A—C80—H80B	109.5
C22—C21—H21	119.7	N2—C80—H80C	109.5
C20—C21—H21	119.7	H80A—C80—H80C	109.5
C23—C22—C21	119.7 (5)	H80B—C80—H80C	109.5
C23—C22—H22	120.2	N2—C81—H81A	109.5
C21—C22—H22	120.2	N2—C81—H81B	109.5
C24—C23—C22	120.3 (5)	H81A—C81—H81B	109.5
C24—C23—H23	119.9	N2—C81—H81C	109.5
C22—C23—H23	119.9	H81A—C81—H81C	109.5
C23—C24—C25	120.5 (5)	H81B—C81—H81C	109.5
C23—C24—H24	119.8		
